# DeepBhvTracking: A Novel Behavior Tracking Method for Laboratory Animals Based on Deep Learning

**DOI:** 10.3389/fnbeh.2021.750894

**Published:** 2021-10-28

**Authors:** Guanglong Sun, Chenfei Lyu, Ruolan Cai, Chencen Yu, Hao Sun, Kenneth E. Schriver, Lixia Gao, Xinjian Li

**Affiliations:** ^1^Department of Neurology of the Second Affiliated Hospital, Interdisciplinary Institute of Neuroscience and Technology, Zhejiang University School of Medicine, Hangzhou, China; ^2^Key Laboratory of Medical Neurobiology of Zhejiang Province, Hangzhou, China; ^3^Key Laboratory of Biomedical Engineering of Ministry of Education, College of Biomedical Engineering and Instrument Science, Zhejiang University, Hangzhou, China; ^4^School of Brain Science and Brain Medicine, Zhejiang University School of Medicine, Hangzhou, China

**Keywords:** movement tracking, behavioral assessment, deep learning, YOLO, background subtraction

## Abstract

Behavioral measurement and evaluation are broadly used to understand brain functions in neuroscience, especially for investigations of movement disorders, social deficits, and mental diseases. Numerous commercial software and open-source programs have been developed for tracking the movement of laboratory animals, allowing animal behavior to be analyzed digitally. *In vivo* optical imaging and electrophysiological recording in freely behaving animals are now widely used to understand neural functions in circuits. However, it is always a challenge to accurately track the movement of an animal under certain complex conditions due to uneven environment illumination, variations in animal models, and interference from recording devices and experimenters. To overcome these challenges, we have developed a strategy to track the movement of an animal by combining a deep learning technique, the You Only Look Once (YOLO) algorithm, with a background subtraction algorithm, a method we label DeepBhvTracking. In our method, we first train the detector using manually labeled images and a pretrained deep-learning neural network combined with YOLO, then generate bounding boxes of the targets using the trained detector, and finally track the center of the targets by calculating their centroid in the bounding box using background subtraction. Using DeepBhvTracking, the movement of animals can be tracked accurately in complex environments and can be used in different behavior paradigms and for different animal models. Therefore, DeepBhvTracking can be broadly used in studies of neuroscience, medicine, and machine learning algorithms.

## Introduction

Behavior measurement and evaluation is one of the key methods to understand brain functions in neuroscience, especially with respect to movement and social behaviors. Different behavior paradigms (e.g., treadmill, open field, y maze, water maze, elevated plus maze, and three-chambered maze) have been developed and used to evaluate the movement, anxiety, social behavior, disease development, sleep disorder, the effect of medication, etc., of an animal (Feng et al., 2020; Yu et al., 2020). More importantly, with technical developments in electrophysiological recording, optical imaging, and optogenetics manipulating in freely behaving animals, we can study brain function in neural microcircuits. To understand the behavior of an animal systematically, it is essential to accurately and quickly quantify the movement of the animal (e.g., direction, speed, distance, and range of motion). However, due to the complexity of laboratory conditions and interference from the camera or other experimental devices used in behavioral recording, it is a significant challenge to track animal locomotion efficiently and precisely.

Given the importance of movement tracking of laboratory animals, numerous open-source programs and commercial systems have been developed for recording and analyzing animal behavior, such as Limelight (Actimetrics, USA) (Jimenez et al., 2018; Ishii et al., 2019; Takemoto and Song, 2019), ANY-maze (Stoelting Co, USA) (Morin and Studholme, 2011; Rodrigues et al., 2019; Feng et al., 2020; Scarsi et al., 2020), Ethovision® XT (Noldus, The Netherlands) (Noldus et al., 2001; Yu et al., 2020), TopScan (Clever Sys Inc., USA) (Grech et al., 2019; Griffiths et al., 2019), Super-maze (Shanghai Xinruan information Technology Co., China) (Hao et al., 2012; Qiao et al., 2017), and others (Samson et al., 2015; Gulyás et al., 2016; Bello-Arroyo et al., 2018; Hewitt et al., 2018). In previous studies, motion tracking in videos captured by the video camera is the most common and low-cost approach to achieve tracking of multiple parameters. Most tracking methods are based on background subtraction algorithms and were developed for rodents. With such an algorithm, an accurate route map can be calculated and drawn based on the contour of targets in a high-definition video image. However, these algorithms are subject to breakdown as both experimental paradigms and laboratory conditions become more complex. In addition, modern technical methods such as electrophysiological recording, optical imaging, and optical stimulation are now widely used with freely behaving animals. High background noise becomes a significant problem, making it difficult to quantify the movement of an animal. The use of background subtraction algorithms alone often cannot effectively separate the target from the high background noise. To overcome these challenges, many alternative methods, such as microwave Doppler radar (Giansanti et al., 2005) and RFID technology (Lewejohann et al., 2009; Catarinucci et al., 2014), have been proposed for tracking animal motion. However, those systems tend to involve additional devices attached to the head of an animal which may be unstable or have a negative influence on the flexibility of the movement of the animal. Also, those systems are expensive and difficult to modify by the user because of high integration and low flexibility.

Recently, researchers in the field of computer vision have advanced a number of algorithms to process image data, including some novel solutions for the detection of moving animals and humans. Some machine learning algorithms have shown high precision in object detection, such as deformable parts models (Felzenszwalb et al., 2010; Unger et al., 2017), R-CNN (Girshick et al., 2014), and deep neural networks (Geuther et al., 2019; Yoon et al., 2019). Using those algorithms, several toolboxes were developed for precisely calculating the postures of laboratory animals during movements, such as DeepLabCut (Mathis et al., 2018; Nath et al., 2019), LEAP (Wang et al., 2017; Pereira et al., 2019), DeepPoseKit (Graving et al., 2019), TRex (Walter and Couzin, 2021), and DANNCE (Dunn et al., 2021; Karashchuk et al., 2021), which greatly simplify and speed up the analysis of multiple behaviors. Although these algorithms may be used to track the gross movement of an animal, they are time-consuming and insufficiently accurate for our purposes because the exact centers cannot be obtained in the process of creating a training dataset.

YOLO is a new generation of deep learning algorithm based on convolutional neural networks (CNN) for object detection (Redmon et al., 2016; Redmon and Farhadi, 2017). Compared with R-CNN, a previous detection algorithm that selects a region of interest (ROI) for possible targets and then identifies targets by classification, YOLO transforms the detection process to a regression problem, predicting the coordinates of the bounding box of the target and classifying the probabilities (*p*-value) of the target directly from the full image through a single network, making it easier to optimize for better performance. Using YOLO, we can predict both the species and locations of experimental animal subjects in a video. However, YOLO only provides the position of an area around the animal (bounding box) instead of the actual position of the animal. The range or position of the bounding box may change abruptly between two sequential frames of the video, even with subtle animal movement. Therefore, it is also difficult to accurately track the position of an animal using only YOLO. Considering the advantages and disadvantages of previous algorithms, we postulated that if YOLO and the background subtraction method were combined, the animal motion could be tracked more accurately and efficiently.

In this study, we propose a laboratory animal behavior tracking method named “DeepBhvTracking” based on both a deep learning algorithm (YOLO) and a background subtraction algorithm. To successfully track animal motion, we first obtain the approximate location of the experimental animal by drawing a bounding box using YOLO, and then, we measure the position of an animal based on the background subtraction algorithm. With our method, movement can be tracked under complex conditions accurately and quickly. All codes with respect to DeepBhvTracking are open-source; the scripts can be customized, and different experimental animal detection models can be easily trained. Overall, DeepBhvTracking is a widely applicable and high-powered behavior tracking method for laboratory animals.

## Materials and Methods

### Materials

All experimental procedures were approved by the Animal Use and Care Committee of Zhejiang University following the National Institutes of Health (NIH) guidelines. Adult wild-type C57BL/6 mice (*n* = 6) were used for most experiments. For movement comparison, mice with *PRRT2* (*n* = 6) and *FMR1* (*n* = 6) mutations were used. Our tracking method was also tested in adult common marmosets (*n* = 3).

For data storage and processing, a high-performance computer (Dell, USA) was used (CPU Intel(R) Core (TM) i7-10700 CPU @ 2.90 GHz, RAM 64 GB, GPU Inter(R) UHD Graphics 630 8 GB). The DeepBhvTracking program was run within MATLAB R2020a with deep learning toolbox, computer vision toolbox, and pretrained deep neural networks: resnet18, mobilenetv2, and resnet50.

In this study, all test videos were taken using a standard webcam (1,080 p for origin videos). For fast processing speed, original videos were read and resized to the resolution of 360^*^640^*^3 pixels for further analysis.

### Methods

#### Model Training

1. Image labeling: Because YOLO is a supervised algorithm, manually labeled images are required for model training (Redmon et al., 2016). To provide training data using a wide range of animal behaviors, images with RGB format were extracted randomly from videos, and a rectangular region around the animal was marked in each image using the Image Label App in the computer vision toolbox of MATLAB. The details of using the Image Label App are available online at https://www.mathworks.com/help/vision/ref/imagelabeler-app.html. After testing under laboratory conditions, we found that around 300 labeled images were usually required for accurate detection under each condition.
2. Image preparation: To test prediction accuracy, the dataset of labeled images was randomly divided into three sets: training (70%), validation (10%), and test (20%). Labeled images in the training set were transformed and resized, including color distortion and information dropping for broad adaptability. The original validation and test sets were retained to evaluate the accuracy of the model.
3. Detector training: In this task, a pretrained deep neural network was transferred and combined with the YOLO algorithm for target detection. For YOLO, the images were normalized to the same resolution for feature extraction. Previous studies indicated that the normalized image size and differences among the pretrained networks have a significant impact on the accuracy and speed of training and tracking. To test this possibility, different pretrained networks including resnet18, mobilenetv2, and resnet50 were tested for each of the following normalized image sizes: 224^*^224^*^3, 320^*^320^*^3, 416^*^416^*^3, and 512^*^512^*^3. In this task, the detector was trained by mini-batch gradient descent (batch size is 16 frames), and the parameters of the network were updated after several iterations via back-propagation. The number of total epochs is 20 and the learning rate is 0.0001. Detailed principles and algorithm derivation follow from previous studies (Redmon et al., 2016; Redmon and Farhadi, 2017). After training, the detector was used for tracking evaluations.

#### Video Tracking

The position of an animal was tracked by combining the deep learning algorithm YOLO with a background subtraction algorithm. Our strategy was to define the bounding box of the target using YOLO and then to obtain the centroid of the target by background subtraction inside the bounding box. Background subtraction tracked moving animals through a pixel-by-pixel comparison of the present image with a background image, as described in detail by others (Barnich and Van Droogenbroeck, 2011).

First, to avoid interference from objects outside the maze, we manually defined the tracking area and set areas outside the tracking area to the background color. Second, the detector trained by YOLO was used to track the position of the animal with a bounding box. In many cases, multiple boxes were detected in one image. In this case, the bounding box with the highest *p*-value was chosen for future use. Next, the bounding box was enlarged 1.5 times to completely cover the whole animal. Finally, a traditional background subtraction method was used to obtain the contour of the animal in the bounding box, and the centroid of the animal was calculated based on the contour.

#### Laboratory Animal Tested by DeepBhvTracking

To evaluate the effectiveness of our tracking method—DeepBhvTracking, black or white mice and marmosets were tested in different behavior paradigms: open field, elevated plus maze, L maze, inverted V-shape maze, and treadmill. We also compared the performance of four tracking methods (background subtraction, YOLO detection, DeepLabCut, and DeepBhvTracking) in three classical behavior paradigms with different noise levels: open field, L maze, and three-chambered maze. Open field is a high signal-to-noise ratio scenario without the interference of wires or operation of an experimenter; in contrast, L maze involves interference from electric wires and hands of the experimenter because of behavior training and calcium imaging. Three-chambered maze is a low signal-to-noise ratio scenario due to the similar color between mice and background. To avoid bias from the training dataset on the performance of different algorithms, 300 images extracted from six videos based on a K-means algorithm were used to train the models of the three deep learning methods: YOLO detection, DeepLabCut, and DeepBhvTracking. In this study, we also compared movement differences among movement-deficit mice (*PRRT2, FMR1*) and wild-type mice. The movement of each animal was recorded for 8 min and tracked by DeepBhvTracking.

#### Comparison With Other Methods

To test tracking efficiency, we compared the training time, tracking speed, error to ground truth, and movement speed of animals detected by the different methods. To compare training speed, both DeepLabCut and DeepBhvTracking models were trained using the same dataset, pretrained neural network (resnet50), and parameters (image number: 300; batch size: 8; iterations: 2000). Tracking speed reflected the video processing speed (frames/second, fps). Error to ground truth was used to estimate the distance between the real location and the estimated location of the target.

### Statistical Analysis

Error bars in all figures represent mean ± SEM, and the number (*n*) of samples employed is indicated in the legends. All data were analyzed by ANOVA followed by LSD test for multiple comparisons, ^*^ indicates *p* < 0.05, ^**^ indicates *p* < 0.01, ^***^ indicates *p* < 0.001.

The codes in this manuscript are open-source, and the detailed list is shown in Table 1. Users can download the codes at GitHub online (https://github.com/SunGL001/DeepBhvTracking).

**Table 1:** Detailed list of codes related to DeepBhvTracking.

**Name**	**Description**
**Training**	
*dbt_dataset*	Get datasets for training
*dbt_training*	Training a detector
*augmentData*	Supporting function used in *dbt_training* for data augmentation
*preprocessData*	Supporting function used in *dbt_training* for resizing image and bounding boxes to the target size
**Tracking**	
*dbt_singleTracking*	Tracking for single file
*dbt_batchTracking*	Tracking for batch files
*dbt_manualTracking*	Manual click tracking undetected and incorrect frames after tracking
*dbt_bhvread*	Supporting function for read video data, supporting for multiple formats
*dbt_createLabeledVideo*	Create the labeled video
*dbt_optimize*	Save undetected and incorrect frames for optimizing model

## Results

### Training and Tracking Optimization

**Figure 1:**
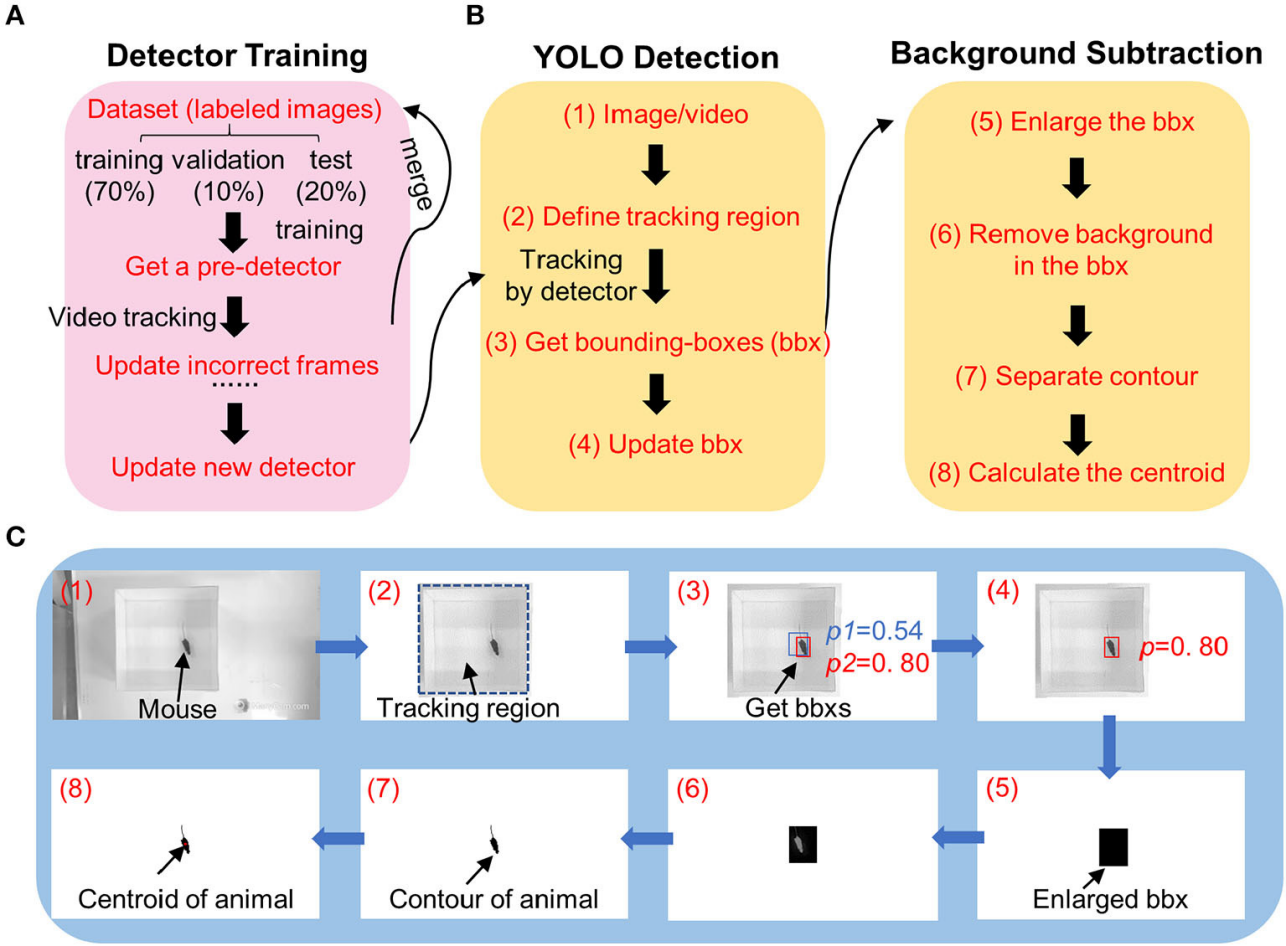
Diagram image showing workflow of DeepBhvTracking. **(A)** Detector training workflow combining a pretrained deep neural network and YOLO algorithm. The parameters were updated via backpropagation. If the detector is not satisfied, wrong images were labeled, and merged into pretrained dataset for detector training again. **(B)** Target tracking workflow. Left, detecting the bounding box using YOLO deep learning method. Right, obtaining centroid with background subtraction method. **(C)** Schematic diagrams showing the tracking process of one frame from an open-field video. The numbers in **C** match the corresponding number in the tracking workflow of **(B)**.

In this task, the position of an animal was tracked by combining pretrained neural networks with the YOLO algorithm and background subtraction (Figure 1). To accurately track the position of different kinds of animals, a well-trained detector was required. We found that the training time was longer, and the tracking accuracy decreased if we trained the model on different kinds of animals together (data not shown). Additionally, we found that the tracking accuracy was highly correlated with the color of the animals and uncorrelated with behavior paradigms. So, we trained the detectors separately for different kinds of animals, namely, white mice, black mice, and marmosets (Supplementary Table 1). Initially, the tracking accuracy was low due to the training dataset that was randomly chosen; those images did not accurately represent the complex postures of the animals. To address this, we added a feedback method to merge undetected images in the training dataset for a better detector. After several iterations, an improved detector was achieved (Figure 1A). We used 1,991 labeled images from different behavior assays for detector training in black mice, 1,458 images in white mice, and 400 images in marmosets (Supplementary Table 1).

During tracking, we found that the deep learning algorithms only provided a bounding box around the target, where the tracking center is the center of the bounding box instead of that of the animal. Moreover, occasionally multiple bounding boxes were obtained or the bounding box did not completely cover the animal, causing the location of the tracking center to change abruptly, resulting in a discontinuous motion trace after analysis. To overcome these limitations, we first detected the bounding box of the target by YOLO at a low threshold. Then, we enlarged the bounding box to completely cover the animal. Third, the contour of the animal was calculated based on background subtraction in the region of the enlarged bounding box (Figures 1B left,C). Last, the centroid of the animal was determined from the center of the contour (Figures 1B right,C).

**Figure 2:**
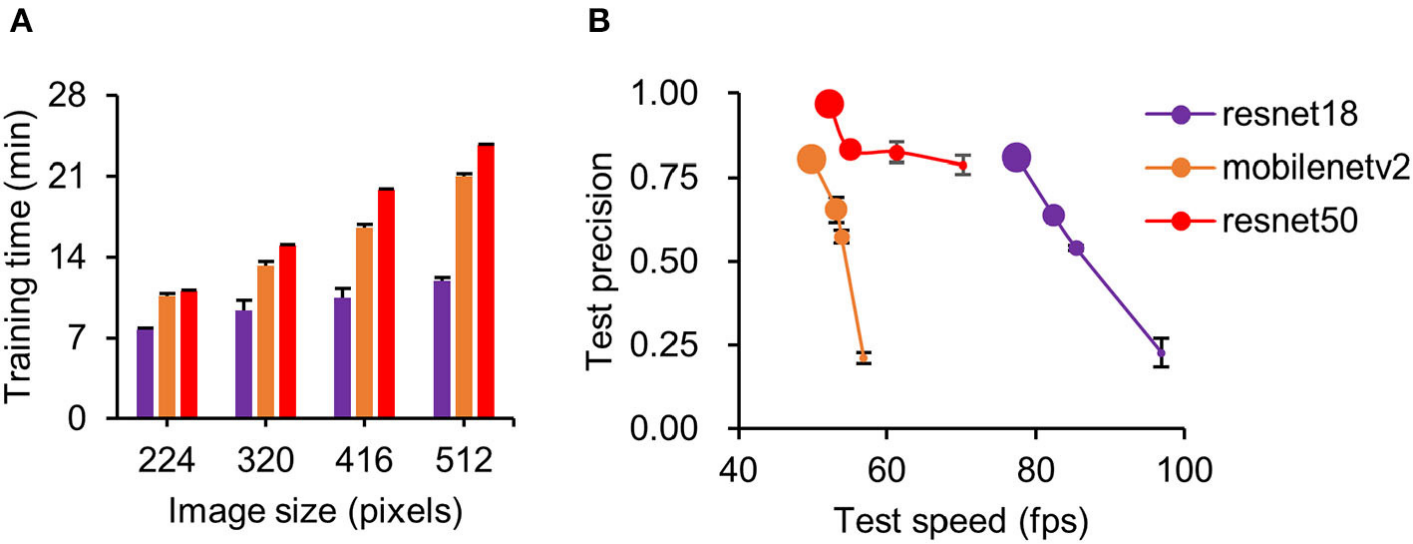
Performance comparison of three pretrained deep neural networks. **(A)** Comparison of training time with different image sizes for three neural networks. Training time increased with image size for all networks. Of the three, resnet50 took the longest time for all image sizes. **(B)** Detection precision plot against training speed for three neural networks at different image sizes. Data point diameter in **(B)** represents the image size from 224 (smallest), 320, 416, and 512 pixels (largest). Note that because of over-fitting, resnet50 is unable to train at an image size of 512 pixels; 480 × 480 images were used instead. Detection precision increased for all networks with increasing image size. In our training model, resnet50 at 480 pixels was used for all conditions.

Three pretrained deep neural networks were evaluated with different image sizes. We found the training time increased with increasing image size (Figure 2A). Of the three neural networks, resnet50 took the longest time at all image sizes (Figure 2A) during detector training (two-way ANOVA, *F* = 360.75, *p* < 0.001). In the detection step, tracking speed (two-way ANOVA, *F* = 92.93, *p* < 0.001) and accuracy (two-way ANOVA, *F* = 197.00, *p* < 0.001) were highly correlated with image size (Figure 2B, see legend). Compared with other networks, resnet50 showed higher precision, and resnet18 showed faster processing speed (Figure 2B). Considering the tradeoff between speed and precision, we set the resnet50 at a resolution of 480^*^480^*^3 pixels (one of the preset parameters used during detector training) as the pretrained deep neural network for constructing our detector. However, resnet18 at 512^*^512^*^3 pixels was also a potentially useful network for simple scenarios (e.g., mice in open-field maze) because it had a high detection speed at relatively high precision. The number of training images used in those three detectors is shown in Supplementary Table 1.

### Smooth Movement Map Tracked by DeepBhvTracking

**Figure 3:**
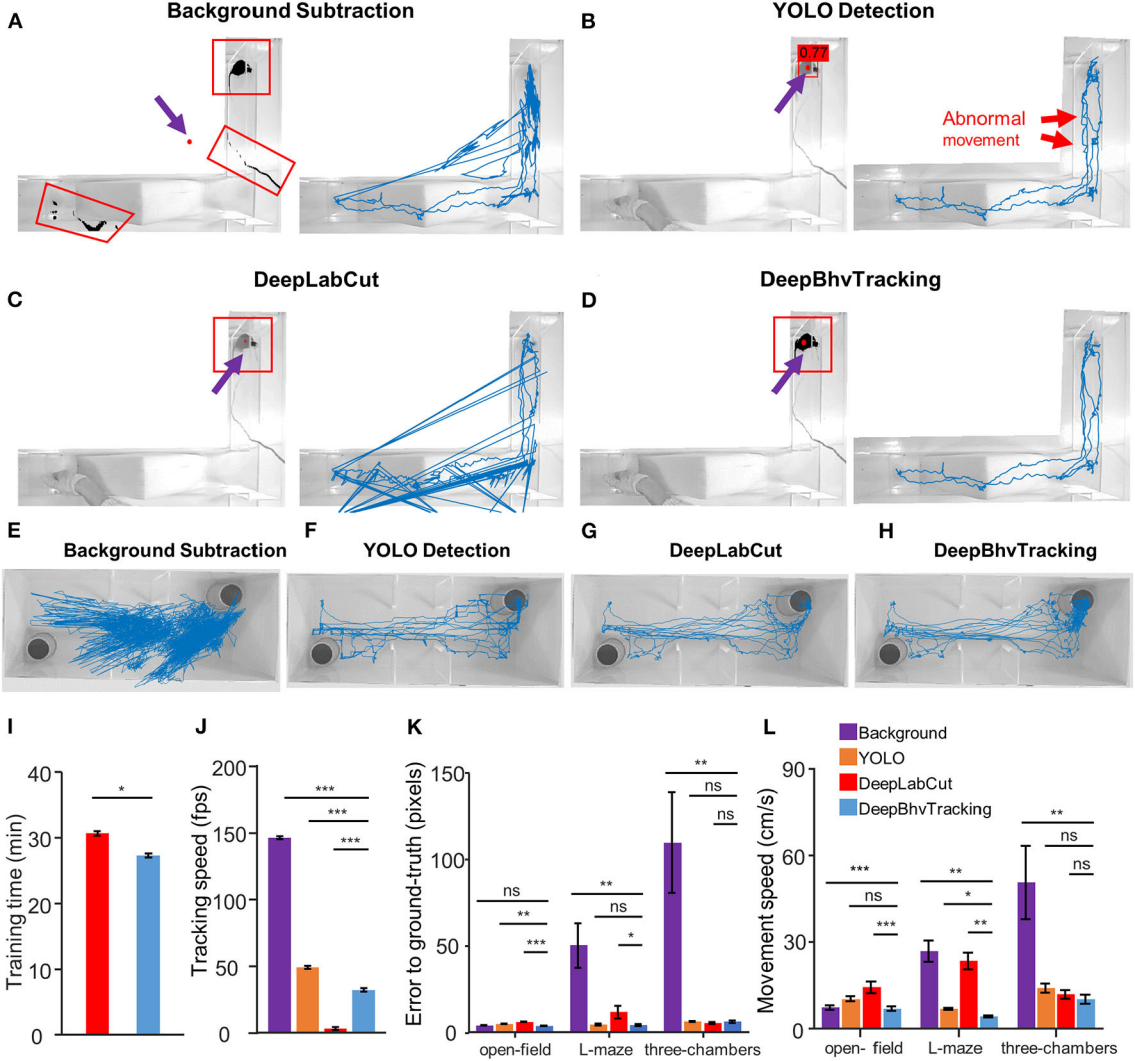
Comparison of three different tracking results for the same video during calcium imaging in a freely behaving mouse. **(A)** Left: Tracking result of one example image with background subtraction only. Black region in red box indicates the detected pixels. Red dot indicates the center of the target. Due to the similar color between the wire and the target animal, the wire has been detected after removing the background. With this method, the target center is marked outside of the tracking area. Right: Blue line represents the resulting movement trace of a mouse in a 5,000 frame video. **(B)** Movement trace tracked by YOLO only. There are many abnormal movements (red arrow) in the tracking trace due to the random jumping of the bounding box. The number above the mouse is the *p*-value predicted by YOLO. **(C)** Movement trace tracked by DeepLabCut. Three of theframes failed to detect according to the trajectory. **(D)** Movement trace tracked by DeepBhvTracking. The movement trace is smooth and represents the actual movement of the animal. **(E–H)** Movement trace in the three-chambered task tracked by background subtraction, YOLO detection, DeepLabCut, and DeepBhvTracking, respectively. **(I)** Comparison of training time between two algorithms. Both models are trained using the same dataset, pretrained neural network, and parameters. Image number: 300; neural network: resnet50; batch size: 8; iterations: 2,000. **(J)** Comparison of tracking time of same video between four algorithms. There are significant differences in processing speed between the four tracking methods. **(K)** Comparison of error to ground truth of four methods in different behavior assays. In these tasks: open field is a simple task without other observable interference; L maze involves wire interference due to the wire cable of free-moving calcium imaging; three-chambered maze involves the condition with a white mouse in bright room light. The sample size for each paradigm is six videos. DeepBhvTracking performed well in all paradigms. The original data are shown in Supplementary Tables 2–4. *p < 0.05, **p < 0.01, ***p < 0.001, tested by ANOVA with LSD test. **(L)** Comparison of movement speed of four methods in different behavior assays.

*In vivo* imaging and electrophysiological recording in freely behaving animals are widely used to understand the neural mechanisms of a particular behavior. Inherent with these techniques are human interference and recording wires that may be captured by the video camera during motion tracking. To address these issues, we compared the tracking accuracy and speed for four tracking methods in three tasks with different kinds of environmental noise (Figure 3). In these tasks, the open field is a simple task without other observable interference; the L maze involves wire and hand image interference due to the wire cable of freely moving calcium imaging; and the three-chambered maze involves conditions with a white mouse in a brightly lit room light which resulted in low contrast between target and environment (Figure 3).

To test training efficiency, we first compared the training time and tracking speed between DeepLabCut and DeepBhvTracking using the same dataset, pretrained neural network (resnet50), and parameters (image number: 300; batch size: 8; iterations: 2000). DeepLabCut showed a slower training speed than YOLO during both training stages (Figure 3I, two-way ANOVA followed by Bonferroni's test, *p* = 0.029) and tracking stage algorithms that are based on deep learning (Figure 3J, two-way ANOVA, *p* < 0.001) with the same computer environments. First, in high background noise conditions, we found that obvious tracking errors were obtained with the background subtraction method alone and that more than 1/10 of frames in one video were required for manual tracking (Figures 3A,E). Using this method alone, the target mouse could be marked outside of the tracking area in some frames due to erroneous calculation of the center based on detected artifacts (Figure 3A). Second, we found that the bounding box of the position of an animal can be easily captured using YOLO with better performance (Figures 3B,F). However, calculating the center of the bounding does not accurately reflect the position of the animal as shown by obviously abnormal motion in the trace (Figures 3B,F, red arrows). Clearly, there are multiple rectangles in the tracking trace which arise from the rapid reorientation of the bounding box. DeepLabCut tracked the center of mice directly and performed well in the three-chambered task (Figure 3G), but there are multiple incorrect frames detected in L-maze which arise from the periodic detection of the hand of the experimenter (Figure 3C). Also, this method cannot exclude systematic error introduced during training dataset preparation (human-defined centroid of the animal). Using DeepBhvTracking, the movement trace is smooth and most accurately represents the actual movement of the animal (Figures 3D,H).

Statistically, compared with DeepLabCut, we found that errors to ground truth, which was used to estimate the distance between the real location and the estimated location of the target, decreased both in the open field (Figure 3K, one-way ANOVA, *F* = 23.93, *p* < 0.001; Figure 3L, one-way ANOVA, *F* = 7.886, *p* = 0.001) and L-maze (Figure 3K, L maze, one-way ANOVA, *F* = 10.70, *p* < 0.001) conditions. Movement speed was lowest in the open field and L maze (Figure 3L, *p* < 0.01) compared with the other three methods. While the YOLO detection algorithm could avoid the interference of wire and hand, the trajectory was not smooth enough because the center of the bounding box does not represent the center of animals (Figures 3B,F,K). DeepLabCut and DeepBhvTracking have similar performances in the three-chambered maze (Figure 3L, LSD test, *p* = 0.871). However, errors to ground truth tracked by DeepLabCut were higher than DeepBhvTracking (LSD test, *p* = 0.040) in the L maze; this may be due to the inaccurate center position of the animal during training dataset construction. In summary, DeepBhvTracking can provide a relatively precise tracking result with fast processing speed in a variety of paradigms (Figure 3).

### Widely Applicable Tracking in Different Paradigms and Animal Models by DeepBhvTracking

**Figure 4:**
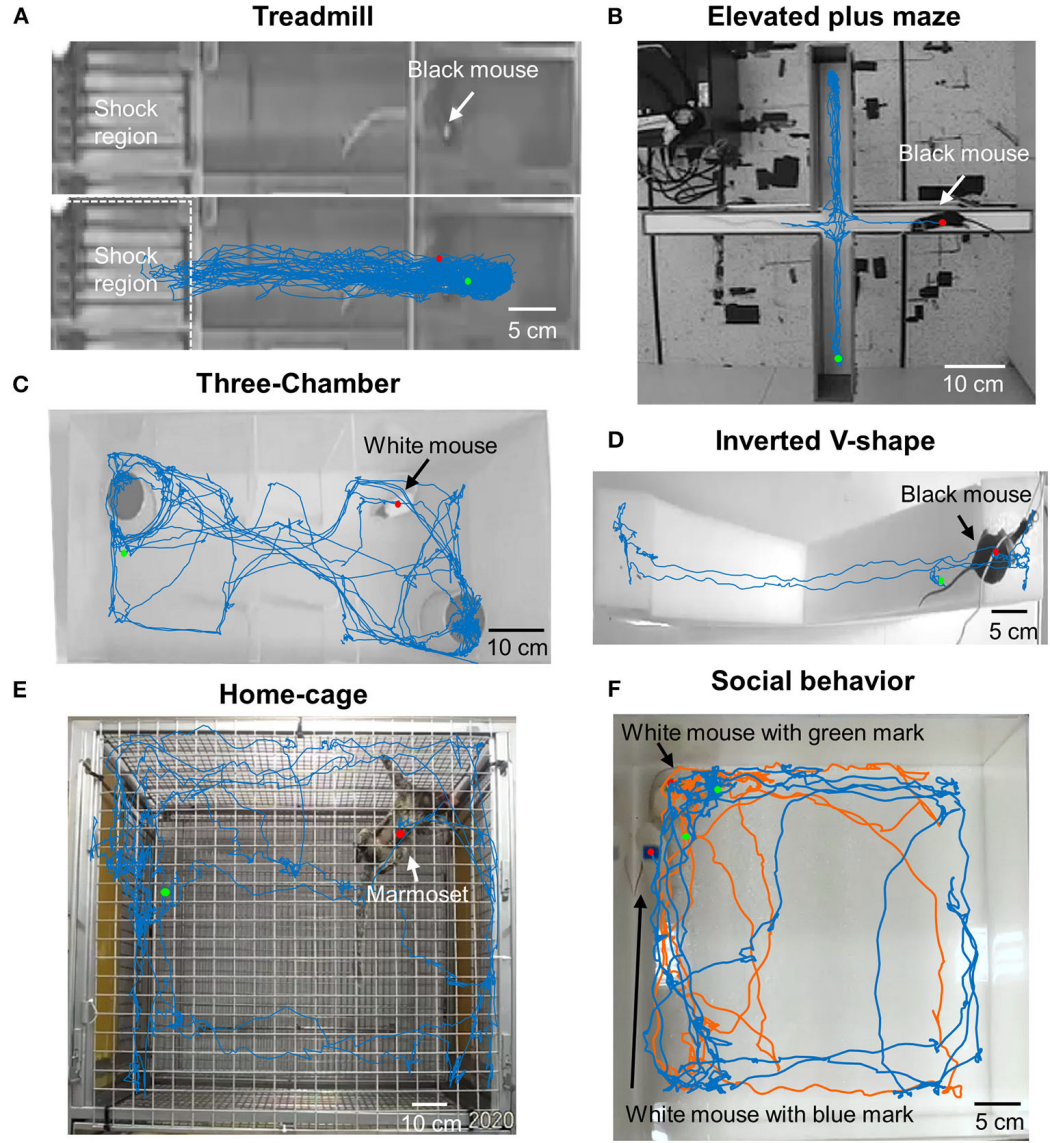
Movement tracking in diverse paradigms using DeepBhvTracking. **(A)** Black mice in treadmill task. With a black mouse on a black background, the ratio between signal and noise is very low. Moreover, the animal only runs in a restricted area because it has been trained not to fall behind the treadmill. It is very difficult to calculate the speed of the animal while performing neuronal decoding in this task. Our DeepBhvTracking method overcomes this challenge and achieves a smooth movement trace. **(B)** Black mice in elevated plus maze. **(C)** White mice in three-chambered box maze. **(D)** Black mice in inverted V-shape maze. **(E)** Marmoset movement trace in home cage. **(F)** Social behavior of white mice in open field. Blue trace: White mouse with a blue mark on its back. Orange trace: White mouse with a green mark on its back.

To check the applicability and flexibility of DeepBhvTracking in different paradigms, black C57BL/6 mice were tested in different environments including open field (Supplementary Figure 1A), L maze (Figure 3C), treadmill (Figure 4A), elevated plus maze (Figure 4B), and inverted V-shape maze (Figure 4D). We obtained smooth movement traces for all conditions (Figures 3C, 4A,B,D; Supplementary Figure 1A). It is worth noting that DeepBhvTracking achieved good performance even in a low target-to-background contrast such as black mice on treadmill (Figure 4A) or white mice in a white environment (Figure 4C). Moreover, in the treadmill assay, animals run only in a restricted area because it will be punished by an electric shock if it falls behind the treadmill. It is usually very difficult to calculate the movement speed of the animal when performing neuronal decoding. Our DeepBhvTracking method overcomes this challenge and achieves a smooth movement trace in treadmill conditions. White mice were trained separately and were tracked in a three-chambered box (Figure 4C) and open field (Figure 4F). Accurate movement tracking was achieved for both conditions. In addition, the movement of marmosets in a 1 m^3^ home cage was tested and a clear movement map was achieved by DeepBhvTracking. Finally, two animals were tracked by labeling each animal with a different color sticker during video recording (Figure 4F blue and orange trace), which indicated our method may also be adapted to social behavior analysis. Hence, DeepBhvTracking is easy and feasible to use with different animal models and different behavior paradigms.

### DeepBhvTracking Can Be Used to Test Movement and Emotion Deficits

The open-field test is one of the most widely used paradigms for assessing locomotor activity and anxiety in rodents. To further test the effectiveness of our tracking method, we performed open-field tests in C57BL/6 wild-type mice and in two widely used movement-deficit mutant animals: *PRRT2* (Chen et al., 2011) and *FMR1* (Baba and Uitti, 2005) (Supplementary Figure 1). For each animal, we first draw out the movement trace of the animal achieved by DeepBhvTracking (Supplementary Figure 1A) and manually checked for no frame losses. Then, spatial pseudo heat maps of movement time (Supplementary Figure 1B) and speed (Supplementary Figure 1C) were calculated. We found that animals stayed longer at the corners than the central area and run faster in the middle of the open field (Supplementary Figures 1B,E). Moreover, both mutants ran faster in the open field than the wild-type animals (Supplementary Figure 1D, one-way ANOVA, *F* = 4.356, *p* = 0.025; Supplementary Figure 1F, two-way ANOVA, *F* = 16.15, *p* < 0.001). Open field can also be used to test the anxiety level base of an animal on the time spent in the corner or center. Based on our tracking method, mice with *PRRT2* mutants stayed at the corner for a shorter time than wild animals (Supplementary Figure 1E, two-way ANOVA, *F* = 18.11, *p* < 0.001). These results may indicate a low anxiety level for *PRRT2* mutants in our open field conditions. Further experiments should be performed to confirm this result.

## Discussion

Accurate behavioral measurement and evaluation are the key steps for pharmacology, neuroscience, and psychological studies. However, commercially available software and open-source programs have many limitations, especially when the experiments are performed under complex environmental conditions. To overcome these difficulties, we designed DeepBhvTracking to track the position of an animal combining deep learning with the YOLO algorithm and background subtraction using the widely used software MATLAB. By incorporating the YOLO detection algorithm, the detection effectiveness is improved by generating a bounding box of the tracked animal (Figures 3B,D,F,H). Simultaneously, background subtraction was applied in the bounding box to acquire an exact location of the animal, which corrects for the slight position deviation inherent in YOLO alone (Figure 3).

We have previously used several commercial software packages: Limelight, ANY-maze, and open-source programs. Although they have superior GUIs, the accuracy is insufficient in dim light and complex environments, and they are confounded by interruption of the recording process, for example, a human hand. Time-consuming manual tracking is required under these circumstances. In addition, the software can only be used under certain predefined conditions and is exceedingly difficult to modify for new environments. Recently, several algorithms, such as DeepLabCut (Mathis et al., 2018; Nath et al., 2019), LEAP (Wang et al., 2017; Pereira et al., 2019), and DeepPoseKit (Graving et al., 2019), based on deep learning have been developed to estimate the posture of an animal during movement. Undoubtedly, those methods can obtain detailed movement information about the targets and have been broadly used in multiple studies (Dooley et al., 2020; Huang et al., 2021). However, these methods have intrinsic limitations to accurate estimation of the centroid of irregular animal targets during training dataset preparation (human-defined center of an animal). Also, tracking speed is very slow with those methods (Figure 3J). Although DeepBhvTracking is also a supervised algorithm, we used background subtraction to correct the systematic error of training dataset preparation. So, DeepBhvTracking is stable, more accurate (Figure 3), less susceptible to background noise, and suitable for different kinds of animals and behavior paradigms (Figure 4). Furthermore, using a feedback training strategy, one can easily improve the detector by adding more labeled images. In addition, DeepBhvTracking also takes advantage of a background subtraction algorithm that defines the centroid of an animal more precisely. With these improvements, we can further increase the tracking accuracy and effectiveness. Finally, DeepBhvTracking is capable of tracking two animals in one video (Figure 4F), if animals are marked with different colors; this indicates that this method is also feasible for the study of social behavior. As a new method, DeepBhvTracking is well-suited to detect multiple types of animals in different scenarios (Figure 4), and it is straightforward to train or optimize the detector according to individual needs. Based on the position tracked by the DeepBhvTracking, the movement distance, the elapsed time, and the speed of the animal can be calculated easily (Supplementary Figure 1).

It is worth noting that DeepBhvTracking can only track the whole-body centroid of an animal; there is no information regarding head direction or body parts. But information of the contour of an animal remains, which makes it possible to define more fine details of an animal, such as the head or tail. In addition, the body parts of animals could be tracked by labeling them with different colors. For example, if we label the nose of a mouse with a red mark and its tail with a green mark, the location of the nose or tail could be tracked by DeepBhvTracking as long as the detector was previously trained to recognize the red and green marks separately.

## Conclusion

We have designed a strategy to track the centroid of an animal combining deep learning with the YOLO algorithm and background subtraction, a tool we call DeepBhvTracking. With this improved method, the motion of laboratory animals can be tracked accurately in a variety of different behavioral paradigms. This in turn offers the potential to speed up many studies in neuroscience, medicine, and so on.

## Data Availability Statement

The data and codes that support the finding of this paper is available at GitHub online (https://github.com/SunGL001/DeepBhvTracking).

## Ethics Statement

The animal study was reviewed and approved by the Animal Experimentation Ethics Committee of Zhejiang University.

## Author Contributions

GS, CL, and RC performed the behavioral recording and finished the data analysis. GS and XL wrote the code. RC, CY, HS, and KS carefully read and edited the manuscript. XL designed the experiments and approved the draft. XL and GS wrote the manuscript. All authors contributed to the article and approved the submitted version.

## Funding

This work was supported by the Natural Science Foundation of China (32071097, 31871056, 61703365, and 91732302), the National Key R&D Program of China (2018YFC1005003), and Fundamental Research Funds for the Central Universities (2019XZZX001-01-20 and 2018QN81008). This work was also supported by the MOE Frontier Science Center for Brain Science & Brain-Machine Integration, Zhejiang University.

## Conflict of Interest

The authors declare that the research was conducted in the absence of any commercial or financial relationships that could be construed as a potential conflict of interest.

## Publisher's Note

All claims expressed in this article are solely those of the authors and do not necessarily represent those of their affiliated organizations, or those of the publisher, the editors and the reviewers. Any product that may be evaluated in this article, or claim that may be made by its manufacturer, is not guaranteed or endorsed by the publisher.

## Supplementary Material

Supplementary Figure 1 Movement for the mice with different genotypes using DeepBhvTracking. **(A)** Example imaging showing the movement of a C57BL/6 mice in open field, the center, and corner regions indicated by the different shaded squares. **(B)** Pseudo heat map showing the animal motion time in **(A)**. **(C)** Pseudo heat map showing animal speed in **(A)**. **(D)** Comparison of average speed among three genotypes of mice. **(E)** Comparison of the time spent in the corner and center among three genotypes of mice. **(F)** Comparison of average speed at the corner and center among three genotypes of mice. ^*^ represents *p* < 0.05, ^**^ indicates *p* < 0.01, tested by the ANOVA with LSD test.

Supplementary Table 1 The number of label images were required for detector training of laboratory animal.

Supplementary Table 2 Comparison of tracking time using four tracking methods in different behavior assays.

Supplementary Table 3 Comparison of pixels change per frame using four tracking methods in different behavior assays.

Supplementary Table 4 Comparison of error to ground-truth using four tracking methods in different behavior assays.
