# Supplementary figures and images for: DeepBhvTracking: A Novel Behavior Tracking Method for Laboratory Animals Based on Deep Learning
Source: Front Behav Neurosci. 2021 Oct 28;15:750894. doi: 10.3389/fnbeh.2021.750894 (PMC8581673; doi:10.3389/fnbeh.2021.750894)

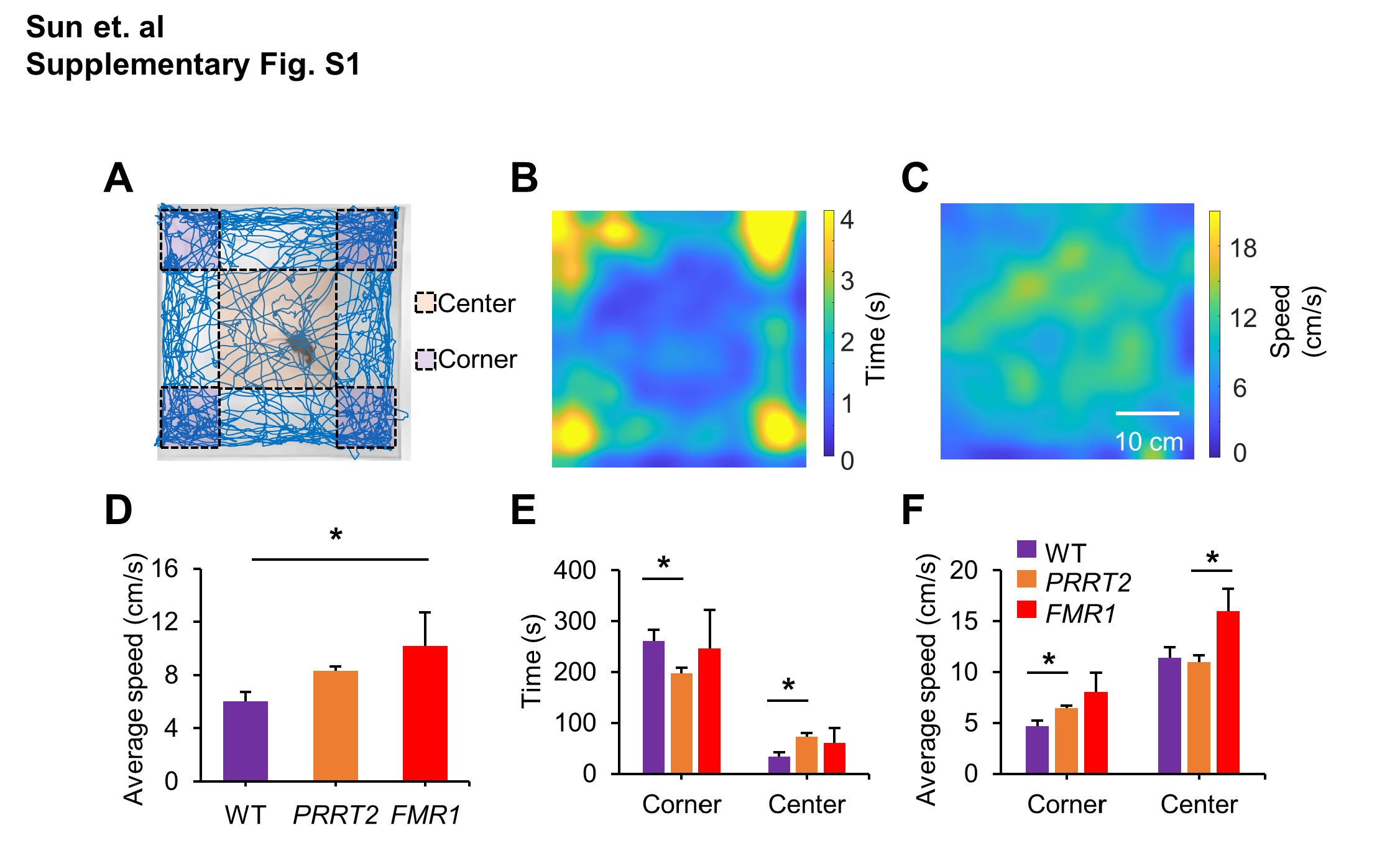

Supplement: Supplementary Figure 1 — Movement for the mice with different genotypes using DeepBhvTracking. (A) Example imaging showing the movement of a C57BL/6 mice in open field, the center, and corner regions indicated by the different shaded squares. (B) Pseudo heat map showing the animal motion time in (A). (C) Pseudo heat map showing animal speed in (A). (D) Comparison of average speed among three genotypes of mice. (E) Comparison of the time spent in the corner and center among three genotypes of mice. (F) Comparison of average speed at the corner and center among three genotypes of mice. * represents p < 0.05, ** indicates p < 0.01, tested by the ANOVA with LSD test. [file Image_1.TIF]
