# Supplementary material for: DeepBhvTracking: A Novel Behavior Tracking Method for Laboratory Animals Based on Deep Learning
Source: Front Behav Neurosci. 2021 Oct 28;15:750894. doi: 10.3389/fnbeh.2021.750894 (PMC8581673; doi:10.3389/fnbeh.2021.750894)
Supplement: Supplementary Table 1 — The number of label images were required for detector training of laboratory animal. [file Table_1.docx]

Table S1. The number of label images were required for detector training of laboratory animal

| Detector | Total | Behavior Assays | | | | | | | | | | |
| --- | --- | --- | --- | --- | --- | --- | --- | --- | --- | --- | --- | --- |
|  |  | open-field maze | L maze | inverted V maze | Y maze | | elevated plus maze | three-chambers maze | | treadmill | | home-cage |
| BlackMice | 1991 | 306+0 | 303+0 | 201+0 | | 312+0 | 306+0 | X | 200+363 | | X | |
| WhiteMice | 1458 | 208+0 | X | X | | X | X | 300+705 | 245+0 | | X | |
| Monkey | 400 | X | X | X | | X | X | X | X | | 400+0 | |

Number before “+” represents the number of images used in pre-detector, number after ‘+” represents the number of images used to reinforce model, X represents the assays which are not tested.

Table S2. Comparison of tracking time using four tracking methods in different behavior assays.

| Animal ID | Background | YOLO | DeepLabCut | DeepBhvTracking |
| --- | --- | --- | --- | --- |
| O1 | 153.74 | 49.45 | 3.30 | 32.07 |
| O2 | 153.31 | 49.33 | 3.34 | 32.15 |
| O3 | 148.78 | 49.39 | 3.30 | 32.43 |
| O4 | 153.98 | 49.86 | 3.30 | 32.62 |
| O5 | 155.37 | 49.75 | 3.30 | 32.66 |
| O6 | 149.58 | 49.77 | 3.29 | 32.74 |
| L1 | 137.62 | 49.68 | 3.36 | 31.39 |
| L2 | 145.75 | 49.48 | 3.41 | 31.55 |
| L3 | 143.52 | 49.36 | 3.24 | 31.59 |
| L4 | 145.91 | 49.38 | 3.31 | 31.79 |
| L5 | 147.53 | 49.34 | 3.30 | 31.85 |
| L6 | 146.34 | 49.43 | 3.31 | 32.01 |
| T1 | 140.85 | 49.11 | 3.23 | 32.19 |
| T2 | 141.38 | 48.46 | 3.23 | 32.96 |
| T3 | 147.45 | 48.81 | 3.21 | 32.91 |
| T4 | 140.58 | 48.55 | 3.19 | 32.84 |
| T5 | 141.83 | 48.75 | 3.20 | 32.37 |
| T6 | 144.80 | 49.64 | 3.20 | 33.94 |
| Mean | 146.57 | 49.31 | 3.28 | 32.34 |
| Se | 1.19 | 0.10 | 0.01 | 0.14 |

The unit is frames per second.

Table S3. Comparison of pixels change per frame using four tracking methods in different behavior assays.

|  | Animal ID | | | | | | Mean | Se |
| --- | --- | --- | --- | --- | --- | --- | --- | --- |
|  | 1 | 2 | 3 | 4 | 5 | 6 |  |  |
| Open field | | | | | | | | |
| Background | 0.65 | 1.42 | 1.45 | 1.04 | 1.32 | 1.46 | 1.22 | 0.12 |
| YOLO only | 1.14 | 1.89 | 2.00 | 1.39 | 1.91 | 2.02 | 1.72 | 0.14 |
| DeepLabCut | 1.24 | 2.71 | 2.19 | 2.66 | 3.58 | 1.99 | 2.40 | 0.29 |
| DeepBhvTracking | 0.52 | 1.37 | 1.41 | 0.97 | 1.30 | 1.42 | 1.17 | 0.13 |
| L maze | | | | | | | | |
| Background | 5.23 | 2.27 | 3.10 | 5.23 | 6.34 | 4.69 | 4.48 | 0.56 |
| YOLO only | 1.08 | 0.97 | 1.01 | 1.40 | 1.18 | 1.26 | 1.15 | 0.06 |
| DeepLabCut | 4.32 | 4.15 | 2.26 | 3.23 | 3.79 | 5.72 | 3.91 | 0.43 |
| DeepBhvTracking | 0.63 | 0.58 | 0.53 | 0.98 | 0.70 | 0.82 | 0.71 | 0.06 |
| Three chambers | | | | | | | | |
| Background | 17.33 | 10.72 | 2.77 | 4.49 | 6.65 | 8.68 | 8.44 | 1.94 |
| YOLO only | 2.77 | 2.19 | 2.48 | 1.24 | 2.45 | 2.96 | 2.35 | 0.23 |
| DeepLabCut | 2.08 | 2.22 | 2.33 | 0.74 | 1.98 | 2.56 | 1.99 | 0.24 |
| DeepBhvTracking | 2.31 | 1.17 | 1.97 | 1.04 | 1.74 | 2.17 | 1.73 | 0.20 |

The unit is pixels.

Table S4. Comparison of error to ground-truth using four tracking methods in different behavior assays.

|  | Animal ID | | | | | | Mean | Se |
| --- | --- | --- | --- | --- | --- | --- | --- | --- |
|  | 1 | 2 | 3 | 4 | 5 | 6 |  |  |
| Open field | | | | | | | | |
| Background | 3.55 | 4.75 | 4.06 | 3.35 | 4.83 | 3.65 | 4.03 | 0.24 |
| YOLO only | 5.03 | 5.52 | 4.94 | 4.47 | 4.63 | 4.94 | 4.92 | 0.14 |
| DeepLabCut | 5.76 | 6.46 | 6.89 | 6.13 | 5.77 | 5.55 | 6.09 | 0.19 |
| DeepBhvTracking | 3.23 | 4.76 | 4.07 | 3.38 | 3.48 | 3.64 | 3.76 | 0.21 |
| L maze | | | | | | | | |
| Background | 19.66 | 61.83 | 75.01 | 24.99 | 95.30 | 25.04 | 50.31 | 11.77 |
| YOLO only | 6.23 | 4.06 | 5.41 | 3.83 | 3.61 | 4.12 | 4.54 | 0.39 |
| DeepLabCut | 30.22 | 8.10 | 10.98 | 7.36 | 7.80 | 5.86 | 11.72 | 3.43 |
| DeepBhvTracking | 7.19 | 4.68 | 4.34 | 3.57 | 2.84 | 3.14 | 4.29 | 0.59 |
| Three chamber | | | | | | | | |
| Background | 84.91 | 24.74 | 196.75 | 38.36 | 139.90 | 173.60 | 109.71 | 26.61 |
| YOLO only | 6.82 | 5.62 | 6.54 | 5.68 | 6.05 | 7.15 | 6.31 | 0.23 |
| DeepLabCut | 6.33 | 3.34 | 6.69 | 4.41 | 5.43 | 6.16 | 5.39 | 0.48 |
| DeepBhvTracking | 9.82 | 3.96 | 7.91 | 6.05 | 4.94 | 6.00 | 6.45 | 0.79 |

The unit is pixels.
